# cRGD peptide-conjugated polyethylenimine-based lipid nanoparticle for intracellular delivery of siRNA in hepatocarcinoma therapy

**DOI:** 10.1080/10717544.2021.1928794

**Published:** 2021-05-27

**Authors:** Shuang Yang, Dandan Wang, Xia Zhang, Yaojun Sun, Bin Zheng

**Affiliations:** a Key Laboratory of Cellular Physiology, Ministry of Education, Shanxi Medical University, Taiyuan, China; b Affiliated Hospital, Changchun University of Chinese Medicine, Changchun, China; c School of Pharmacy, Shanxi Medical University, Taiyuan, China

**Keywords:** Targeting peptide, nanoparticles, polyethyleneimine, siRNA, antitumor

## Abstract

The effective delivery system plays an important role in the application of siRNA in the antitumor study. However, until now, researches on the delivery systems targeting hepatocarcinoma cells are still being explored. Here we designed and prepared a novel siRNA delivery system, cRGD-PSH-NP, which was based on a modified polyethyleneimine (PSH) and DSPE-PEG_2000_-cRGD. cRGD-PSH-NP loaded with survivin siRNA (cRGD-PSH-NP/S) was composed of egg phosphatidylcholine, cationic PSH, PEGylated lipids, survivin siRNA, and cRGD peptide as a targeting ligand. The formulations of cRGD-PSH-NP/S were optimized and characterized. *In vitro* investigations showed excellent gene silencing and antitumor activity compared with the unmodified nanoparticles in HepG2 cells. *In vivo* antitumor efficacy of cRGD-PSH-NP/S exhibited potent tumor inhibition (74.71%) in HepG2-bearing nude mice without inducing toxicity. These data suggested further research of cRGD-PSH-NP/S in hepatocarcinoma therapy.

## 1. Introduction

Hepatocarcinoma is one of the most common tumors with high mortality all around the world. Chemotherapy is a common treatment. However, the survival of the patients is dismal (Wu et al., 2020; Yang & Heimbach, 2020). RNA interference (RNAi) technology provides challenges and therapeutic opportunities for hepatocarcinoma (Hajiasgharzadeh et al., 2019), because RNAi can make intracellular mRNA-specific degradation occurs, leading to silencing of target gene expression (Bhattacharjee et al., 2019; Setten et al., 2019). It has been confirmed that survivin is highly expressed in hepatocellular carcinoma, so survivin is a potential target of RNAi for the treatment of hepatocarcinoma (Su, 2016). In recent years, more and more researches have focused on the preclinical research of small interfering RNA (siRNA) of cancer (Scarabel et al., 2017; Subhan & Torchilin, 2019). As a kind of nucleic acid drug, siRNA showed rapid clearance in the circulation and poor transport across biological barriers which resulting in limited treatment efficiency (Kulkarni et al., 2019). So effective delivery systems are promising to protect siRNA and be capable to release siRNA into the cytoplasm (Barba et al., 2019; Dong et al., 2019).

During the past years, many efforts have been made to construct efficient nano-drug delivery systems (Khan et al., 2019). Suitable delivery systems are promising to have the appropriate size and electric charge, meanwhile, they should have high biocompatibility and low toxicity to cells (Han et al., 2019). The controllable release of siRNA is also a difficult point in the design of the delivery systems (Ma, 2014; Mainini & Eccles, 2020). To improve the biocompatibility of the delivery systems, many kinds of nanocarriers were used such as liposomes (Diao et al., 2019), microemulsions (Wang et al., 2019), nanocapsules (Jiang et al., 2018), and cationic nanoparticles (Jin et al., 2019). It is noteworthy that intracellular environment-sensitive delivery systems are efficient and controllable for the special microenvironment of tumor tissue (Kozielski et al., 2014). In recent years, redox-responsive nanoparticles have received tremendous attention for triggered siRNA release in response to the higher concentration of reductive matters (7- to 10-fold) in tumor cells compared to that in normal cells (Pezzoli et al., 2016; Chi et al., 2017). In order to further improve the accumulation of siRNA in the tumor cells, targeting components, such as receptor-recognizing peptides and antibodies, which were connected to various species of nanoparticles. It was reported that a targeting peptide, cyclic arginine-glycine-aspartic acid (cRGD)-modified nanoparticle, enhanced the tumor accumulation through the targeting of α_v_β_3_ integrin, which is over expressed in various types of malignant tumors such as melanoma, glioblastoma, hepatocarcinoma, breast, pancreatic and ovarian carcinoma (Cheng & Ji, 2019).

In our previous study, a reduction-sensitive cationic polymer (PEI-SS-HA, PSH) was synthesized and the nanoparticle contained PSH was designed to deliver an antisense oligonucleotide, which showed suitable properties and high transfection efficiency (Zheng et al., 2019). In order to further design a system with safety, low toxicity, high siRNA loading efficiency, hepatocarcinoma targeting, and rapid intracellular drug release, here we report a multifunctional nanoparticle based on logical design. Egg phosphatidylcholine (ePC), cholesterol, DSPE-PEG were incorporated into a supported bilayer. Cationic PSH was incorporated into the nanoparticle to give the ability of reduction-sensitive and the ‘proton pump effect’ based on PEI to release siRNA into the nucleus rapidly. In order to increase circulation time and target the hepatocarcinoma cells precisely, DSPE-PEG_2000_-cRGD was used to constitute the outermost structure as a targeting ligand of the nanoparticles. The properties of the nanoparticles were characterized, the *in vitro* and *in vivo* transfection activity was evaluated.

## 2. Materials and methods

### 2.1. Materials

Egg phosphatidylcholine (ePC), cholesterol, and DSPE-PEG_2000_ were purchased from Avanti Polar Lipids, Inc (Shanghai, China). DSPE-PEG_2000_-cRGD was purchased from Xi’an Ruixi Biological Technology Co., Ltd (Xi’an, China). Survivin siRNA (5′-mGCAGGUUCCUmUAUCUGUCATT-3′) and 5′-Cy3 labeled survivin siRNA were synthesized by Biomics Biotechnologies (Jiangsu, China). PEI (branched, 25 kDa) and hexadecyl amine were purchased from Sigma-Aldrich (St. Louis, MO, USA). Dulbecco’s modified Eagle’s medium (DMEM), Roswell Park Memorial Institute 1640 (RPMI 1640), and fetal bovine serum (FBS) were purchased from HyClone (Logan, UT, USA). Anti-survivin, anti-GAPDH antibody and horseradish peroxidase (HRP)-conjugated goat anti-rabbit IgG were obtained from Abcam Inc. (Cambridge, MA, USA). HepG2 cells and LO2 cells were purchased from American Type Culture Collection (ATCC). All other analytical reagents were commercially obtained in reagent grade.

### 2.2. Preparation and optimization of the formulation of the nanoparticles

**Figure 1.**
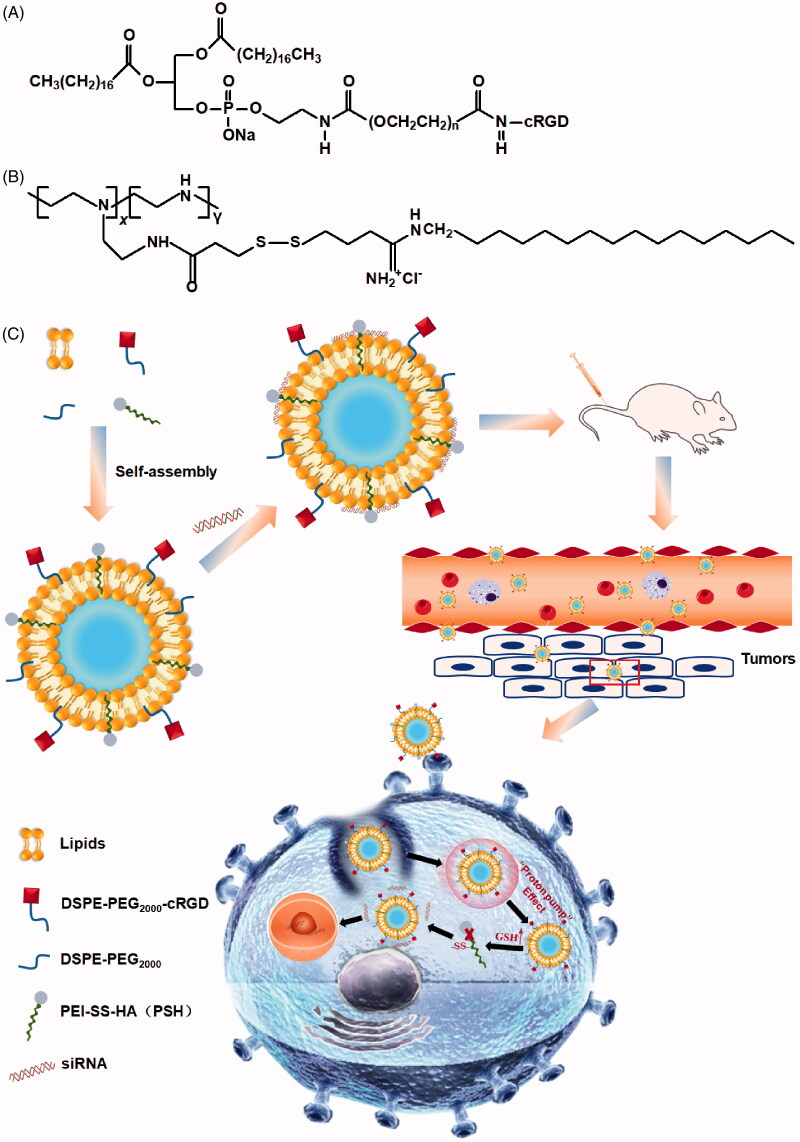
The structural formulae of DSPE-PEG_2000_-cRGD, PSH and schematic diagram of the structure of cRGD-PSH-NP and its application for siRNA delivery to cancer cells. (A) Structural formulae of DSPE-PEG_2000_-cRGD. (B) Structural formulae of PSH. (C) Schematic diagram of the structure of cRGD-PSH-NP and its application for siRNA delivery to cancer cells.

PEI-SS-HA (PSH) was synthesized as described in our previous report (Zheng et al., 2019). The structural formulae of DSPE-PEG_2000_-cRGD and PSH were showed in Figure 1(A,B).

The ethanol dilution method was used for preparing the lipid nanoparticles (NP) or the lipid nanoparticles containing survivin siRNA (NP/S). In order to verify that cRGD-PSH-NP was superior to other nanoparticles as a carrier of siRNA, PEI-NP, PSH-NP, and cRGD-NP were prepared and used to combine with survivin siRNA. Briefly, for the NP containing PSH (PSH-NP), ePC, cholesterol, PSH, and DSPE-PEG_2000_ were dissolved in ethanol with homogeneous mixing to obtained the lipid solution, the molar ratios of PSH/total lipids were from 10% to 50%. The lipid solution was then injected into PBS solution (pH 6.8) under rapid mixing at the volume ratios of 1:9 to obtain PSH-NP with different concentrations of PSH. For the PSH-NP containing siRNA (PSH-NP/S), survivin siRNA was dissolved in pre-cooled DEPC water and added into the PSH-NP solution followed by mixing for 60 s at various PSH/siRNA molar ratios from 12:1 to 2:1. Meanwhile, PEI-NP/S was prepared as the group of control to confirm the low toxicity and high cellular uptake efficiency of modified PEI (PSH), and to prove the necessity of adding PSH instead of PEI into nanoparticles. In the process of preparation, PSH was is replaced by PEI (25 kDa) in the group of PEI-NP/S, other processes were consistent with the preparation method of PSH-NP/S. For the NP containing cRGD (cRGD-NP), ePC, cholesterol, PEI, DSPE-PEG_2000_, and DSPE-PEG_2000_-cRGD were dissolved in ethanol, which was injected into PBS solution (pH 6.8) under rapid mixing. Survivin siRNA was then dissolved in pre-cooled DEPC water and added into the cRGD-NP followed by mixing for 60 s to obtain cRGD-NP/S. cRGD-NP/S was used for the comparison to show whether PSH could increase the transfection efficiency in nanoparticles. Finally, cRGD-PSH-NP, which represented the NP containing cRGD and PSH was designed and prepared. Briefly, ePC, cholesterol, PSH, DSPE-PEG_2000_, and DSPE-PEG_2000_-cRGD were dissolved in ethanol. Survivin siRNA was then dissolved in the obtained solution at different molar ratios of cRGD/total lipids from 1% to 9%, the final system was named cRGD-PSH-NP/S. The diagram of the composition and drug release process *in vivo* of cRGD-PSH-NP/S is shown in Figure 1(C).

The particle size and zeta potential of PSH-NP at various PSH/total lipids molar ratios, PSH-NP/S at various PSH/siRNA molar ratios and cRGD-PSH-NP/S at different molar ratios of cRGD/total lipids were determined on a Zetasizer Nano ZS 90 instrument (Malvern Instruments, Ltd., Malvern, UK). Each sample was measured averaging three times. Meanwhile, the morphology of cRGD-PSH-NP/S was observed under the field emission scanning electron microscope (FE-SEM) (JSM-6700F, JEOL, Tokyo, Japan) at 3.0 kV accelerating voltage.

### 2.3. Determination of the nanoparticles combined with siRNA by gel retardation assay

PSH-NP/S at various ratios of PSH:siRNA (12:1 to 2:1) were prepared and confirmed by agarose gel retardation assay to investigate the ability of PSH-NP to form nanocomplexes with survivin siRNA. Then PEI-NP/S, PSH-NP/S, cRGD-NP/S, and cRGD-PSH-NP/S were prepared at the optimal formulation and confirmed by agarose gel retardation assay. Ten microliters of the samples were incubated with 10× sample buffer and then loaded onto a 2% agarose gel containing 5% (v/v, μl/ml) Goldview. After electrophoresis (120 V, 15 min), the gels were photographed under UV-illumination.

The stability of siRNA and cRGD-PSH-NP/S was investigated by agarose gel retardation assay. siRNA and cRGD-PSH-NP/S were mixed with equal-volume fetal bovine serum (FBS) and incubated at 37 °C for 2, 4, 6, 8, 10, and 12 h, respectively. TritonX-100 was added into the solution until the final concentration was 1%, and undegradable siRNA was released for electrophoresis and image development. The same amount of siRNA was used as control.

### 2.4. Cell culture

HepG2 cells and LO2 cells were propagated in DMEM and RPM1640 supplemented with 10% FBS and 1% antibiotics (100 units/ml penicillin and 100 μg/ml streptomycin), respectively. The cells were cultured at 37 °C in a humidified 5% CO_2_ incubator.

### 2.5. Cytotoxicity assay of the nanoparticles

HepG2 cells and normal liver cells LO2 were seeded at a density of 1 × 10^4^ cells/well in a 96-well plate for 24 h. Cells were treated with PEI-NP, PSH-NP, cRGD-NP, or cRGD-PSH-NP at various concentrations for 4 h, the medium was removed and incubated in fresh DMEM or RPM1640 medium for another 20 h. Then each well was incubated with 20 μl MTT (5 mg/ml in PBS solution) stock solution for 4 h. The culture medium was removed and then 100 μl/well of DMSO was added to dissolve formazan crystals that formed, the absorbance was measured at OD 490 nm in a plate reader.

### 2.6. In vitro treatment and cellular uptake of the nanoparticles

The cellular uptakes of 5′-Cy3-labeled survivin siRNA which were wrapped in nanoparticles were determined by an EPICS XL flow cytometer (Beckman Coulter Corp., Tokyo, Japan) in HepG2 cells. Specifically, HepG2 cells were plated on 24 well plates (1 × 10^5^ cells/well) and cultured for 24 h in DMEM medium with 10% FBS. Then, the cells were washed three times with PBS solution and a fresh serum-free DMEM medium was added. Then cells were treated with naked siRNA, PSH-NP/S, cRGD-NP/S, or cRGD-PSH-NP/S for 4 h without or with FBS at 37 °C, the siRNA was labeled with 5′-Cy3 in the above component. The localization and the quantitative fluorescence intensity of the nanoparticles after uptaking by cells were realized by tracking the fluorescence signal of Cy3. Then the HepG2 cells were washed with PBS solution three times and fixed in 4% paraformaldehyde. The cells were collected and analyzed by flow cytometry and untreated HepG2 cells were collected as a negative control.

The internalizations of the nanoparticles were further observed by Zeiss 710 LSMNLO Confocal Microscope (Carl Zeiss; Jena, Germany). HepG2 cells were cultured in a glass-bottom cell culture dish (1 × 10^5^ cells/well) for 24 h. Naked siRNA, PSH-NP/S, cRGD-NP/S or cRGD-PSH-NP/S were added into the cells and incubated without or with FBS for 4 h at 37 °C, the siRNA was labeled with 5′-Cy3. Then the cells were washed three times with PBS solution and fixed in 4% paraformaldehyde for 15 min. Cellular nuclei were stained with DAPI (3 μg/ml) for 3 min. The cells were washed with PBS solution three times to remove excess dye.

### 2.7. Determination of the survivin protein expression

HepG2 cells were plated on 6 well plates (1 × 10^5^ cells/well) overnight. Then, the medium was removed and cells were treated with PEI-NP/S, PSH-NP/S, cRGD-NP/S, or cRGD-PSH-NP/S for 4 h at 37 °C. Cells were cultured for another 44 h with a fresh medium. RIPA buffer containing 2% PMSF and 1% protease inhibitor cocktail was added to lyse the cells for 10 min at 4 °C. The lysed solution was collected and centrifuged at 10,000 rpm for 5 min to obtain the total proteins. Bradford protein assay was used to determine the concentration of protein. Then 40 μg total proteins were separated by 10% SDS-PAGE gel and transferred onto polyvinylidene fluoride (PVDF) membrane. The PVDF membrane was blocked with 5% bovine serum albumin for 3 h and immunoblotted against GAPDH or survivin antibody at 4 °C overnight. The membrane was then incubated with HRP-conjugated goat anti-rabbit IgG for 4 h at 4 °C. ECL detection kits (GE Healthcare Life science, Waukesha, WI, USA) were used for detecting chemiluminescence.

### 2.8. In vivo antitumor efficacy

To investigate the *in vivo* antitumor activity of the nanoparticles, HepG2 cells were transplanted into BALB/C nude mice. The nude mice were randomly divided into three treatment groups with three mice: the control group (normal saline), cRGD-NP/S group, and cRGD-PSH-NP/S group. When the tumor volume reached approximately 100 mm^3^, the nanoparticles were injected via the tail vein. Briefly, the control group was injected with 0.2 ml 0.9% saline solution, the cRGD-NP/S group or cRGD-PSH-NP/S group was injected with 0.2 ml cRGD-NP/S or cRGD-PSH-NP/S (2.5 mg/kg siRNA per injection). The drug loading of each batch of nanoparticles was determined by agarose gel retardation assay before administration. Triton X-100 was added to the nanoparticle solution until the final concentration was 1%, siRNA was released and agarose gel retardation assay was performed to calculate the drug loading before administration. All the groups were dosed every 3 days for 21 days. The size of the tumor and the bodyweight were monitored every 3 days. The antitumor activity was evaluated in terms of tumor volume (*V*), which was estimated by the equation *V* = *a* × *b*^2^/2, where *a* and *b* are the largest and smallest diameter of the tumor, respectively. At the same time, weights of mice were taken. After the last treatment, the xenograft tumors were harvested and photographed, and the protein levels of survivin in tumors were analyzed by Western blot.

### 2.9. In vivo safety evaluation

After continuous injection for 21 days, euthanasia was conducted on all mice and major organs including heart, liver, spleen, lung, and kidney were collected and fixed in 4% paraformaldehyde, sliced, and then stained with hematoxylin and eosin (H&E). Images of tissue sections were detected by ImageXpress^®^ Pico (Molecular Devices Corporation, CA, USA).

### 2.10. Statistical analysis

All the data in the above experiments were expressed as mean ± standard deviation (SD). The significant differences between different groups were carried out with *t*-test. *p* < .05 represents a significant difference, and *p* < .01 indicates a highly significant difference.

## 3. Results

### 3.1. Formulation optimization of cRGD-PSH-NP/S

**Figure 2.**
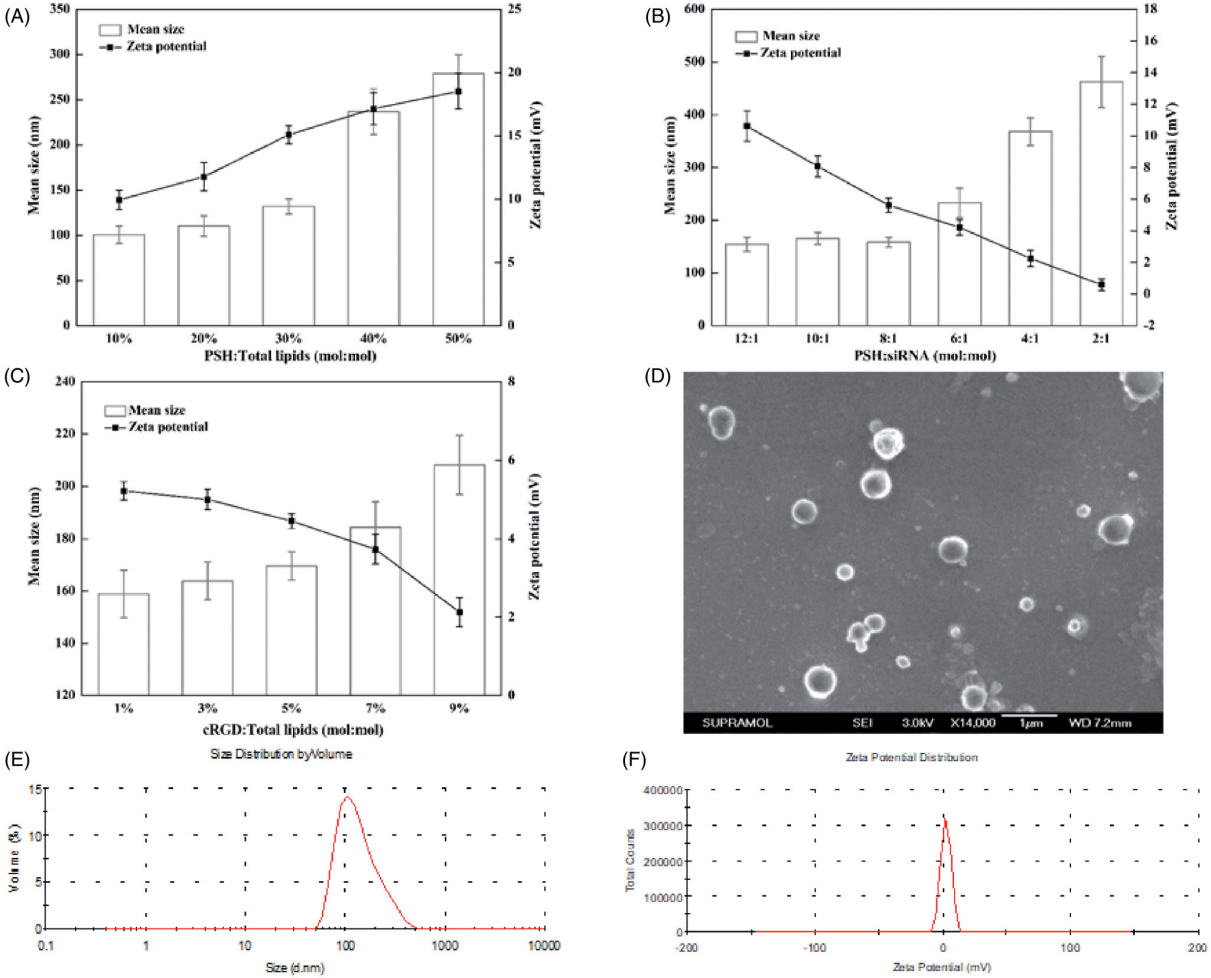
Formulation optimization and characterization of cRGD-PSH-NP/S. (A) Mean particle sizes and zeta potentials of PSH-NP at various PSH/Total lipids molar ratios from 10% to 50%. (B) Mean particle sizes and zeta potentials of PSH-NP/S at various PSH/siRNA molar ratios from 12:1 to 2:1. (C) Mean particle sizes and zeta potentials of cRGD-PSH-NP/S at various cRGD/Total lipids molar ratios from 1% to 9%. (D) SEM image of cRGD-PSH-NP/S at cRGD/Total lipids molar ratio = 5%. (E) Diameter distribution of cRGD-PSH-NP/S at cRGD/Total lipids molar ratio = 5%. (F) Zeta potential distribution of cRGD-PSH-NP/S at cRGD/Total lipids molar ratio = 5%.

The particle sizes and zeta potentials of PSH-NP with various concentrations of PSH were firstly measured to explore the appropriate concentration of PSH (Figure 2(A)). As the ratios of PSH:total lipids increased, both the particle sizes and zeta potentials gradually increased. When the ratio of PSH:total lipids was greater than or equal to 40%, the standard deviations of both particle sizes and zeta potentials became obviously larger. So too much PSH was not conducive to the formation of nanoparticles. When the ratio of PSH:total lipids was 30%, the particle size, and zeta potential were 131.87 ± 8.45 nm and 15.09 ± 0.70 mV, which were both suitable for encapsulating siRNA.

**Figure 3.**
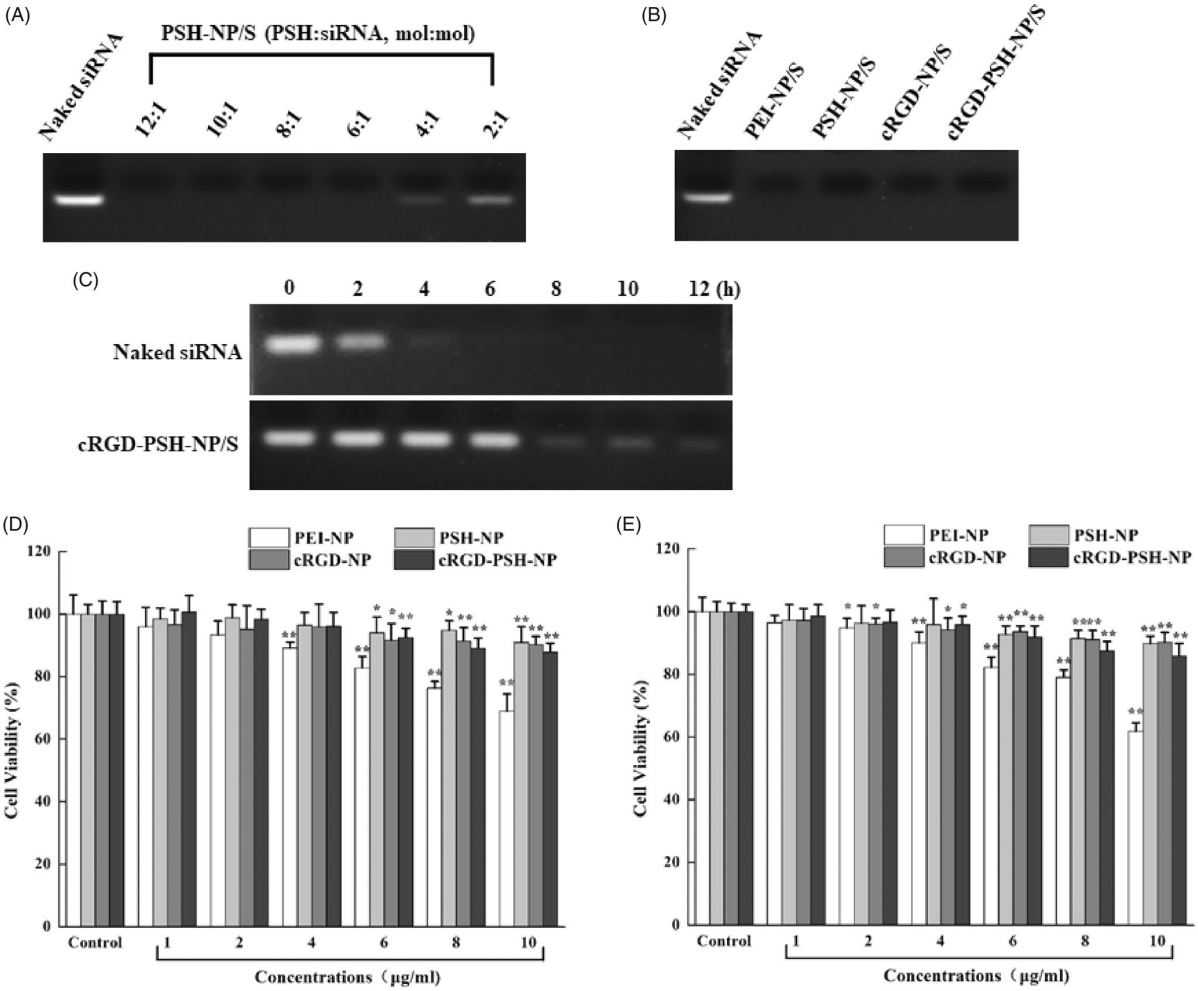
Agarose gel retardation assay and cytotoxicity tests of the nanoparticles. (A) Electrophoretic bands of PSH-NP/S at various PSH/siRNA molar ratios from 12:1 to 2:1. (B) Electrophoretic bands of PEI-NP/S, PSH-NP/S, cRGD-NP/S and cRGD-PSH-NP/S. (C) Electrophoretic bands of naked siRNA and cRGD-PSH-NP/S after incubating with FBS for several time periods. (D) Cell viability of HepG-2 cells treated with PEI-NP, PSH-NP, cRGD-NP and cRGD-PSH-NP. (E) Cell viability of LO2 cells treated with PEI-NP, PSH-NP, cRGD-NP and cRGD-PSH-NP. Each bar is the mean of six experiments normalized to mean ± SD. (*p < .05 vs control; **p < .01 vs control).

In order to load siRNA efficiently, the ratio of siRNA in nanoparticles was necessary to be controlled. As shown in Figure 2(B), the change of particle size was not obvious when the value of PSH:siRNA was 12:1 to 8:1. Unfortunately, as the value of PSH:siRNA changed to 6:1 or less, the particle size increased obviously. Meanwhile, as the concentration of siRNA increased, the zeta potential decreased. In order to further verify the combination of PSH-NP and siRNA at various ratios of PSH:siRNA (12:1 to 2:1), agarose gel retardation assay was used. Figure 3(A) showed that the bright electrophoretic band disappeared as the ratio of PSH:siRNA increased, which showed that PSH-NP could completely combine with siRNA when the ratio of PSH:siRNA was greater than 6:1. Hence, PSH:siRNA = 8:1 was the appropriate ratio for the preparation of PSH-NP/S from the result of the agarose gel retardation assay, which was consistent with the result of particle size and zeta potential measurement.

The concentration of the targeting peptide had an important influence on the physical and chemical properties of the nanoparticles. For the group of cRGD-PSH-NP/S, the particle sizes and zeta potentials of the nanoparticles with various concentrations of cRGD were tested (Figure 2(C)). As the ratio of cRGD:total lipids changed from 1% to 9%, the zeta potentials showed decreased slightly, which might result from the presence of aspartic acid in cRGD. However, with the gradual increase of cRGD, the particle sizes gradually increased, possibly due to the agglomeration of nanoparticles. Especially, when the ratio of cRGD:total lipids was greater than 5%, the particle size of cRGD-PSH-NP/S increased obviously. Based on the above optimization results, the optimal formulation of cRGD-PSH-NP/S was as follows: PSH:total lipids = 30%, PSH:siRNA = 8:1, cRGD:total lipids = 5%.

### 3.2. Characterization of cRGD-PSH-NP/S

FE-SEM was used to observe the morphology and size of cRGD-PSH-NP/S. The SEM image was shown in Figure 2(D). The shape of cRGD-PSH-NP/S was mostly spherical and the surface was smooth. The size distribution of cRGD-PSH-NP/S was 100–400 nm within the field of view under SEM. The results of the particle size and the zeta potential of cRGD-PSH-NP/S measured by the laser particle size analyzer were shown in Figure 2(E,F), the mean values were 169.60 ± 5.43 nm and 4.45 ± 0.19 mV.

In order to investigate the combination of cRGD-PSH-NP and the other nanoparticles with survivin siRNA, an agarose gel retardation assay was implemented. As shown in Figure 3(B), naked survivin siRNA showed bright electrophoretic bands, and the electrophoretic bands of PEI-NP/S, PSH-NP/S, cRGD-NP/S, and cRGD-PSH-NP/S disappeared, which showed PEI-NP, PSH-NP, cRGD-NP, and cRGD-PSH-NP could completely combine with survivin siRNA at the tested ratios. PEI-NP and PSH-NP could bind to siRNA, cRGD-NP, and cRGD-PSH-NP could still bind tightly to siRNA after adding DSPE-PEG_2000_-cRGD, there was no separation of the vectors and siRNA within a certain concentration range.

In order to investigate the stability of cRGD-PSH-NP/S in FBS, an agarose gel retardation assay was conducted. As shown in Figure 3(C), the electrophoretic band of the naked siRNA group disappeared completely after incubating with FBS for 6 h, while the electrophoretic band of the cRGD-PSH-NP/S group could still be seen for 12 h. The result showed that cRGD-PSH-NP could effectively protect siRNA from being degraded by nucleases in serum.

### 3.3. Cytotoxicity tests

Excellent nanoparticles loaded with siRNA should be safe and have low toxicity, so MTT assay was used to test the viability of HepG2 and LO2 cells after they were treated with PEI-NP, PSH-NP, cRGD-NP, or cRGD-PSH-NP, the results were shown in Figure 3(D,E). For the group of PEI-NP, the cell viability of HepG2 cells and LO2 cells decreased gradually with the increase of the concentration of PEI-NP. PEI-NP showed toxicity because of the large numbers of aminos. Fortunately, PSH-NP, cRGD-NP, and cRGD-PSH-NP showed lower toxicity compared with PEI-NP. The cell viability of HepG2 and LO2 cells remained above 80% at the experimental concentrations, the addition of the nanoparticles did not have major impacts on cell viability, which showed PSH-NP, cRGD-NP, and cRGD-PSH-NP were safe as the vehicles for delivering survivin siRNA.

### 3.4. Cellular uptakes of the nanoparticles

**Figure 4.**
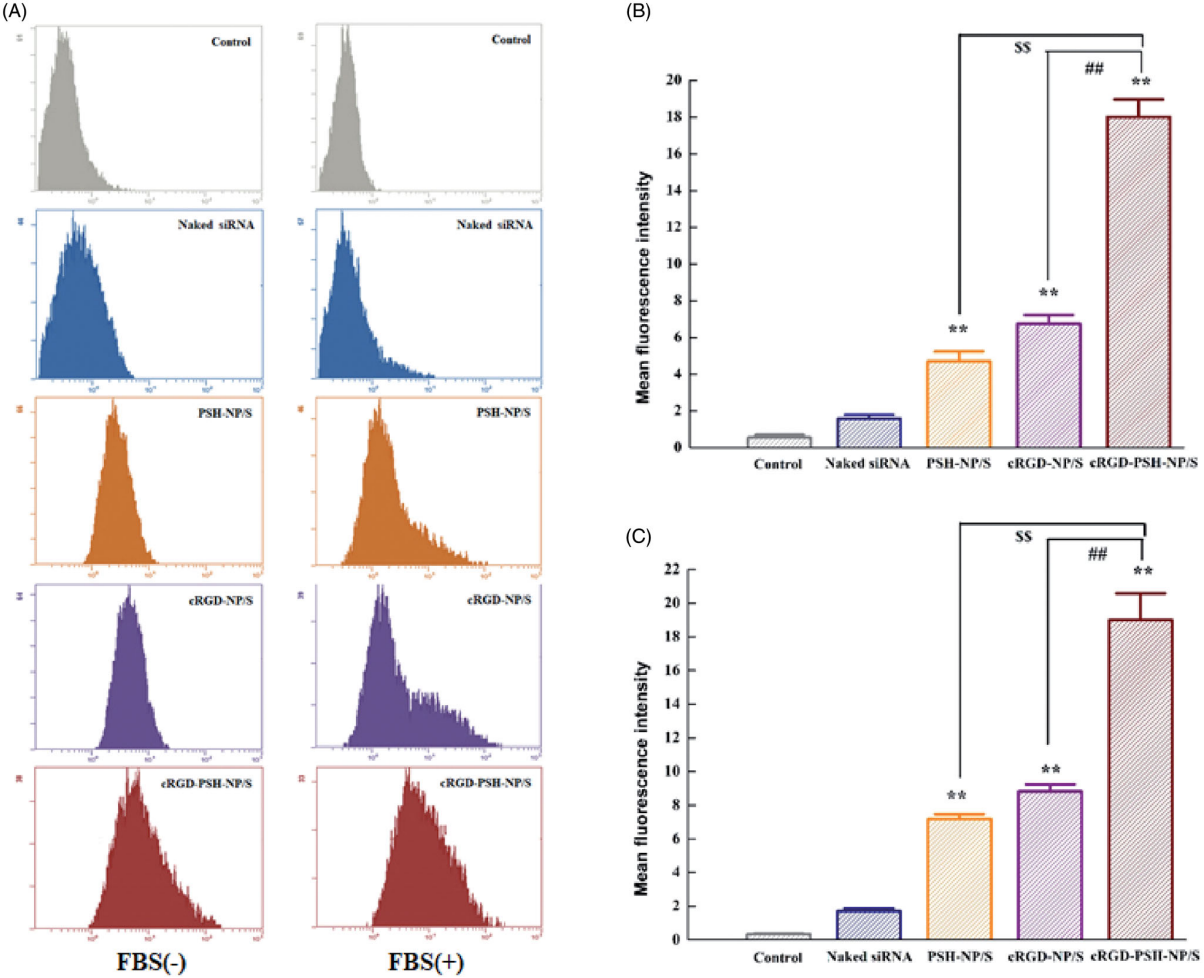
Cellular uptake of 5′-Cy3-labled surviving siRNA delivered by the nanoparticles. (A) Cellular fluorescence uptake *in vitro* by flow cytometry in HepG2 cells without and with FBS. (B) The mean fluorescence values of HepG2 cells treated with naked siRNA, PSH-NP/S, cRGD-NP/S and cRGD-PSH-NP/S without FBS. (C) The mean fluorescence values of HepG2 cells treated with naked siRNA, PSH-NP/S, cRGD-NP/S and cRGD-PSH-NP/S with FBS. Each bar is the mean of three experiments normalized to mean ± SD. (**p < .01 vs naked siRNA; ^$$^*p* < .01 vs PSH-NP/S; ^##^*p* < .01 vs cRGD-NP/S).

The cellular uptakes of 5′-Cy3-labeled survivin siRNA delivered by PSH-NP, cRGD-NP and cRGD-PSH-NP in HepG2 cells were firstly determined by flow cytometry. Figure 4(A) showed the fluorescence migration curve of HepG2 cells treated with naked siRNA, PSH-NP/S, cRGD-NP/S, or cRGD-PSH-NP/S. The migration diagram on the left showed that no FBS involved in the administration process, and the one on the right had FBS. The fluorescence curve of the group of naked siRNA did not significantly migrate compared with the control group because the siRNA was degraded by nuclease or repelled from the cell membrane due to the strong negative charge. By contrast, the curves of PSH-NP/S, cRGD-NP/S, and cRGD-PSH-NP/S were significantly migrated, especially the group of cRGD-PSH-NP/S with or without FBS, which showed that in the presence of FBS, cRGD-PSH-NP could also efficiently transfect survivin siRNA into HepG2 cells. The mean fluorescence intensities of HepG2 cells were presented to evaluate the transfection efficiency of each group more specifically (Figure 4(B,C)). The mean fluorescence intensity values of the group of cRGD-PSH-NP/S were 18.00 ± 0.96 and 19.03 ± 1.57 in HepG2 cells with or without FBS, which were more than 2.5 times that of cRGD-NP/S and 3 times that of PSH-NP/S. For the cells treated with cRGD-PSH-NP/S, the mean fluorescence intensity was significantly increased compared with PSH-NP/S and cRGD-NP/S (*p* < .01), whether FBS existed or not. The results of cellular uptakes determined by flow cytometry of the nanoparticles showed cRGD-PSH-NP was effective in improving the transfection efficiency of survivin siRNA.

### 3.5. Intracellular distribution of the nanoparticles by confocal laser microscopy

**Figure 5.**
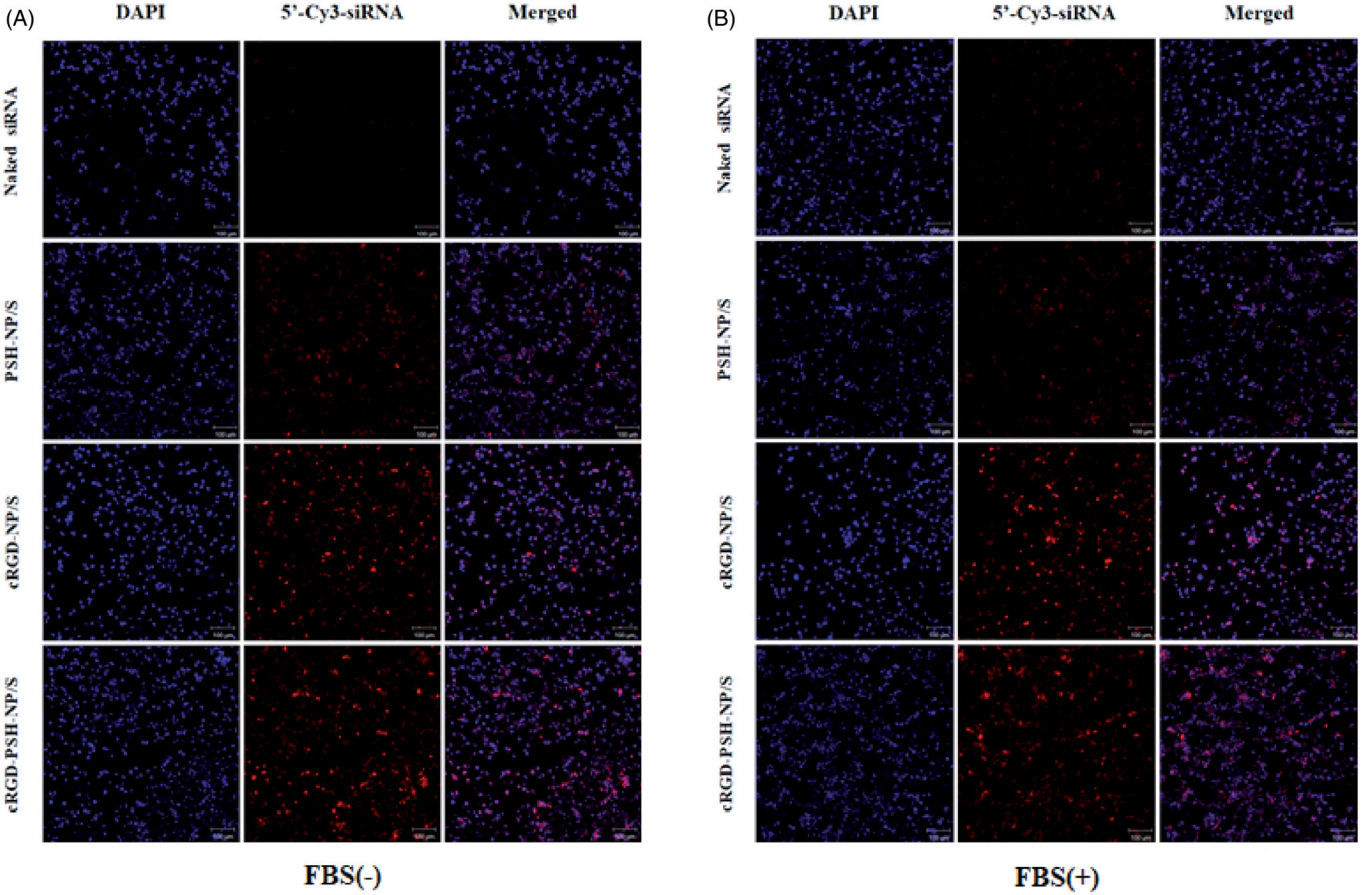
Intracellular distribution of the nanoparticles by confocal laser microscopy. (A) Intracellular localization of 5′-Cy3-labled siRNA delivered by PSH-NP, cRGD-NP and cRGD-PSH-NP without FBS in HepG2 cells (10×). (B) Intracellular localization of 5′-Cy3-labled siRNA delivered by PSH-NP, cRGD-NP and cRGD-PSH-NP with FBS in HepG2 cells (10×). 5′-Cy3-labled siRNA is shown in red, DAPI nuclear stain is shown in blue. Scale bar = 100 μm.

Confocal laser microscopy was also used to observe the transfection of naked siRNA, PSH-NP/S, cRGD-NP/S, and cRGD-PSH-NP/S more directly, siRNA was labeled with 5′-Cy3. Figure 5(A,B) showed the intracellular distribution of naked siRNA, PSH-NP/S, cRGD-NP/S, and cRGD-PSH-NP/S in HepG2 cells without FBS and with FBS, respectively. The images showed that only weak red fluorescence in the field of vision of the group of naked siRNA which indicated naked siRNA could not effectively close to the cell membrane and then internalize into the cell to perform RNAi without the help of the vectors. And as expected, the red fluorescence signals of the groups of PSH-NP/S, cRGD-NP/S, and cRGD-PSH-NP/S were enhanced in the field of vision compared with the naked siRNA, especially the group of cRGD-PSH-NP/S. Results showed the uptake of survivin siRNA by HepG2 cells was obviously enhanced with the help of cRGD-PSH-NP even in the presence of FBS.

**Figure 6.**
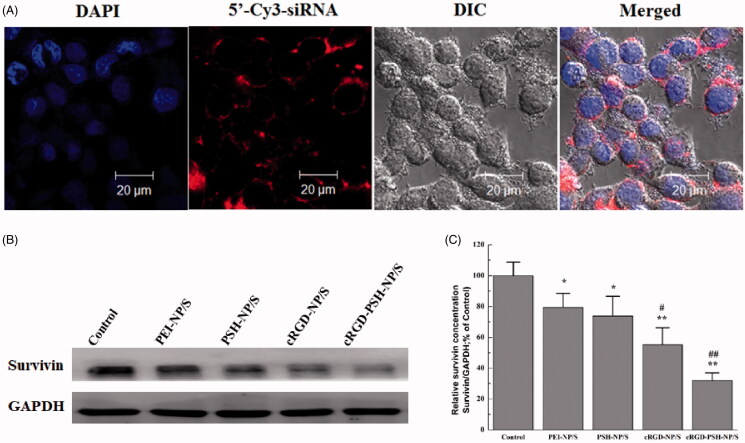
The internalization of cRGD-PSH-NP/S under high magnification (63×) and the down regulation of survivin protein expression in HepG2 cells. (A) Intracellular localization of 5′-Cy3-labled siRNA delivered by cRGD-PSH-NP/S under high magnification (63×) with FBS. (B) The down regulation of survivin protein expression in HepG2 cells treated with PEI-NP/S, PSH-NP/S, cRGD-NP/S and cRGD-PSH-NP/S (*p < .05 vs control; **p < .01 vs control; ^#^*p* < .05 vs PEI-NP/S; ^##^*p* < .01 vs PEI-NP/S).

Figure 6(A) showed the internalization of cRGD-PSH-NP/S under high magnification (63×). Within the field of view, a lot of red fluorescence was observed around or inside the blue nucleus, which showed 5′-Cy3-labeled siRNA delivered by cRGD-PSH-NP entered HepG2 cells in large quantities, cRGD-PSH-NP/S could cross the cell membrane and be distributed in the cytoplasm. The results of the confocal laser microscope in Figure 6(A) further illustrated that cRGD-PSH-NP was an effective vector for loading and delivering survivin siRNA in HepG2 cells.

### 3.6. Determination of survivin protein expression

The expression level of survivin protein was an important indicator to evaluate whether the above vectors transfect siRNA into HepG2 cells efficiently. The survivin protein levels in HepG2 cells treated with PEI-NP/S, PSH-NP/S, cRGD-NP/S, or cRGD-PSH-NP/S were determined by Western blot, the image and analysis result were shown in Figure 6(B,C). The levels of survivin protein in PSH-NP/S, cRGD-NP/S, and cRGD-PSH-NP/S groups were all down-regulated at varying degrees, especially of the group of cRGD-PSH-NP/S. More specifically, the relative survivin protein expression of PEI-NP/S, PSH-NP/S, cRGD-NP/S, and cRGD-PSH-NP/S were 79.42%, 73.83%, 55.23%, and 32.01%, respectively. Correspondingly, the survivin down-regulations were 20.58%, 26.17%, 44.77%, and 67.99%, respectively. The survivin down-regulation of cRGD-PSH-NP/S in HepG2 cells was significantly improved compared with the other groups, which showed the adornment of cRGD and PSH was effective for increasing the transfection efficiency of siRNA in HepG2 cells.

### 3.7. *In vivo* antitumor efficacy of the nanoparticles

**Figure 7.**
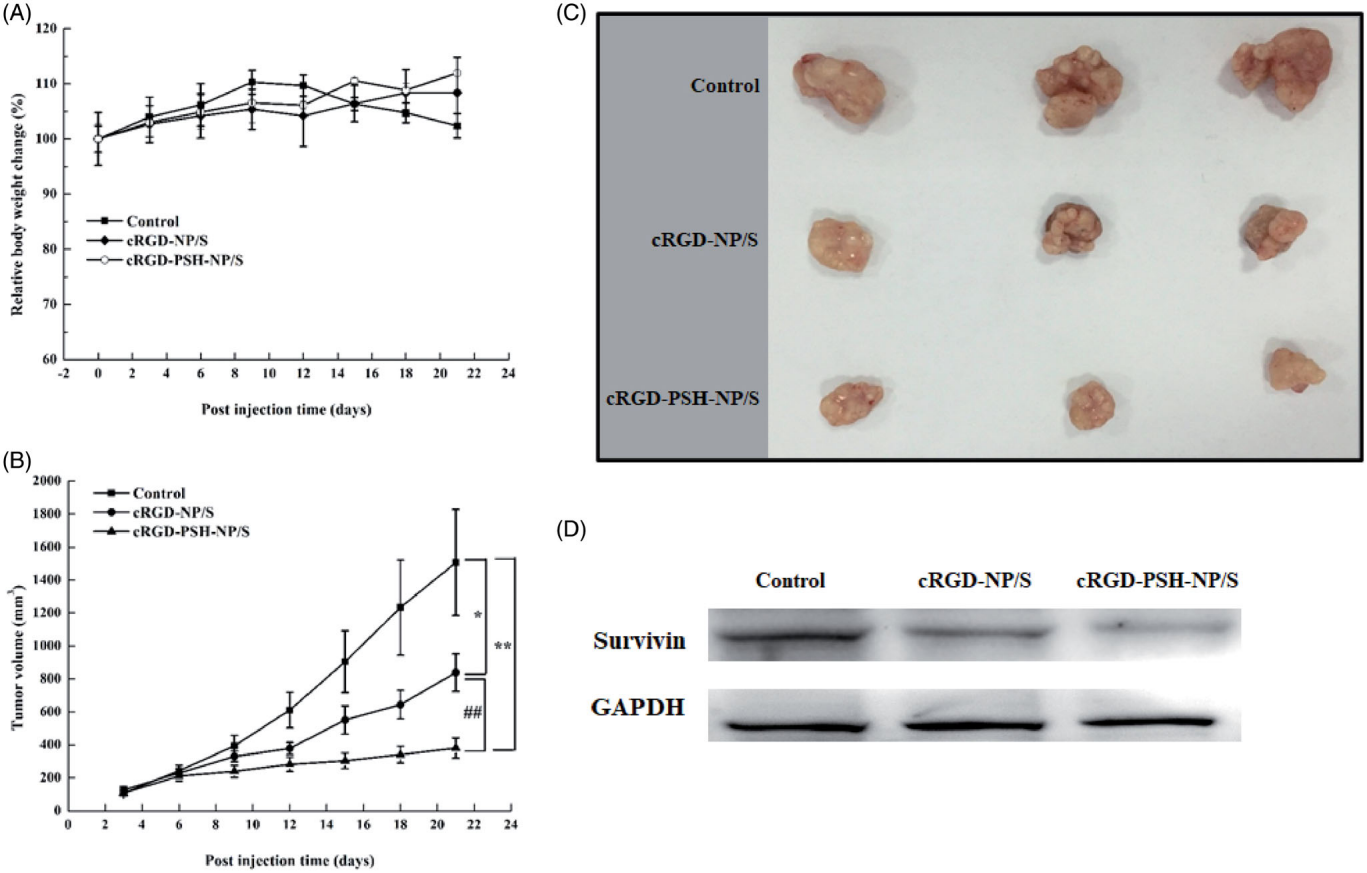
*In vivo* antitumor efficacy of the nanoparticles. (A) The relative body weight change of nude mice in the process of treatment. (B) The curves of the tumor volume of xenograft tumors in nude mice (*p < .05 vs control; **p < .01 vs control; ^##^*p* < .01 vs cRGD-NP/S). (C) Photographs of xenograft tumors harvested at the end of treatment (*n* = 3). (D) The level of survivin analyzed by Western blot in tumors treated with cRGD-NP/S and cRGD-PSH-NP/S.

*In vivo* antitumor efficacy of cRGD-NP/S and cRGD-PSH-NP/S were evaluated in BALB/C nude mice. The relative body weights and the tumor volumes are shown in Figure 7(A,B). During the administration period, the weight of the nude mice did not decrease significantly in the group of cRGD-NP/S and cRGD-PSH-NP/S, however, since the 12th day of administration, the weight of the nude mice in the control group decreased, which was closely related to the rapid growth of the tumors. The tumor growth curve after administration demonstrated that the tumor growth in cRGD-NP/S and cRGD-PSH-NP/S groups was significantly slower than that in the normal saline-treated control group. It was obvious that the cRGD-NP/S and cRGD-PSH-NP/S inhibited the tumor growth effectively, especially the group of cRGD-PSH-NP/S. The tumor inhibition rates of cRGD-NP/S and cRGD-PSH-NP/S were 44.30%, and 74.71%, respectively. The inhibition rate of cRGD-PSH-NP/S was significantly higher than that of the saline-treated control (*p* < .01) and of cRGD-NP/S (*p* < .01). The photograph of the harvested tumors further confirmed the tumor inhibition (Figure 7(C)) and verified cRGD-PSH-NP/S was effective for delivering survivin siRNA into HepG2 cells. Meanwhile, there was no obvious functional abnormality in eating, behavior, response to external stimulus in the group of cRGD-NP/S and cRGD-PSH-NP/S. Further, the Western blot analysis result showed that the expression levels of survivin in tumors treated with cRGD-NP/S and cRGD-PSH-NP/S were lower than that of the control group, and the level of survivin in tumors treated with cRGD-PSH-NP/S was significantly lower than that treated with cRGD-NP/S (Figure 7(D)). These results confirmed that cRGD and PSH could help the siRNA enter tumor cells and enhance the efficiency of RNAi in tumor-bearing mice.

### 3.8. In vivo safety evaluation

**Figure 8.**
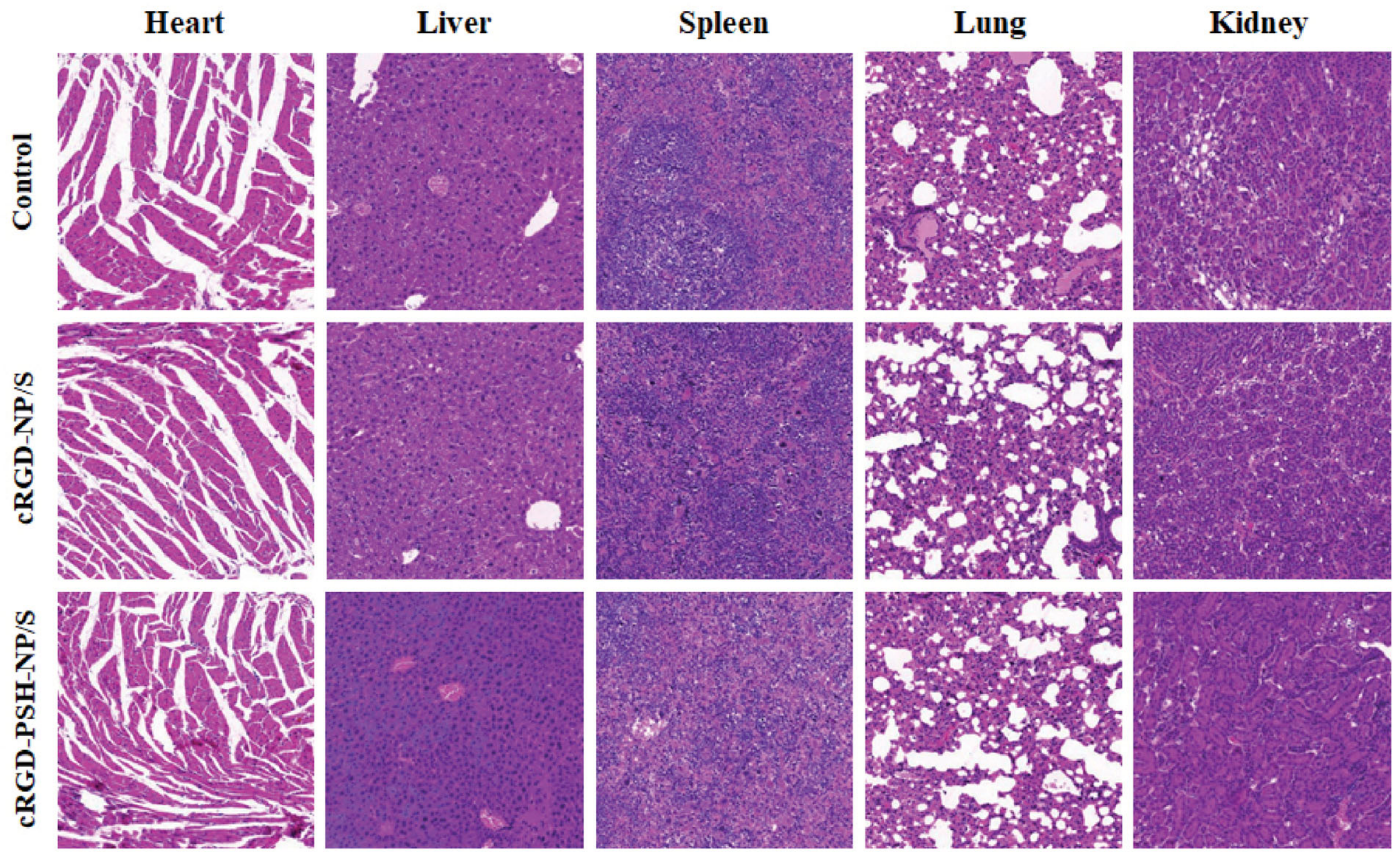
*In vivo* safety evaluation of the nanoparticles. Hematoxylin and eosin (H&E) staining images (40×) of tissue sections from mice after the continuous injection of normal saline, cRGD-NP/S and cRGD-PSH-NP/S for 21 days. Tissues were harvested from heart, liver, spleen, lung and kidney.

The *in vivo* safety of cRGD-NP/S and cRGD-PSH-NP/S in mice was further evaluated through H&E staining. There were no obvious histological differences between the major organs including heart, liver, spleen, lungs, and kidney of cRGD-NP/S, cRGD-PSH-NP/S, and saline-treated mice (Figure 8). Images proved that the major organs did not show any significant damage or inflammation to the mice in the groups of cRGD-NP/S and cRGD-PSH-NP/S.

## 4. Discussion

As a potential nucleic acid drug, siRNA needs efficient transport vectors to assist it to exert the RNA interference effect (Khalil et al., 2018; Jiao et al., 2020). In this study, a reduction-sensitive cationic polymer PSH and a targeting ligand cRGD were used for preparing the nanoparticle named cRGD-PSH-NP to deliver survivin siRNA (Figure 1). The formulations and characteristics of cRGD-PSH-NP loaded with siRNA (cRGD-PSH-NP/S) were investigated, meanwhile a series of the *in vitro* and *in vivo* transfection activity were evaluated.

The results of the formulation optimization of cRGD-PSH-NP/S showed the molar ratios of various components of cRGD-PSH-NP/S had an important influence on its particle size and zeta potential. Excessive PSH might cause instability or even aggregation of nanoparticles (Yang et al., 2019). The additional siRNA could cause the nanoparticles to approach neutrality and thus decrease the interaction with the cell membrane. DSPE-PEG_2000_-cRGD could form a dense structure on the surface of nanoparticles which will help to prolong the circulation time in the blood and provide targeting ligand, however, excessive DSPE-PEG_2000_-cRGD could also make the nanoparticles unstable. Finally, based on the results of particle size, zeta potential, and agarose gel retardation assay, the optimized molar ratios of various components were: PSH:total lipids = 30%, PSH:siRNA = 8:1, and cRGD:total lipids = 5% (Figure 2(A–C), 3(A)). The particle size and zeta potential of cRGD-PSH-NP/S prepared by the optimal formulation were 169.60 ± 5.43 nm (<200 nm) and 4.45 ± 0.19 mV, under this condition (Figure 2(E,F)), which were suitable for enhanced permeability and retention effect of solid tumor. Other studies on the properties of the nanoparticles show that cRGD-PSH-NP could bind tightly to siRNA (Figure 3(B)) and cRGD-PSH-NP could effectively protect siRNA from being degraded by nucleases in serum (Figure 3(C)). The cell viabilities of HepG2 and LO2 cells treated with PSH-NP, cRGD-NP, and cRGD-PSH-NP remained above 80% at the experimental concentrations in MTT assay, which proved PSH and DSPE-PEG_2000_-cRGD based on PEI and cRGD had been modified to reduce toxicity. The above information showed cRGD-PSH-NP were safe as the vehicles for survivin siRNA in HepG2 cells and normal liver cells (Figure 3(D,E)).

The related experiments *in vitro* showed that the cellular uptakes of 5′-Cy3-labeled survivin siRNA delivered by cRGD-PSH-NP in HepG2 cells were significantly increased compared with PSH-NP/S and cRGD-NP/S, which were 3 times that of PSH-NP despite the presence of FBS (Figure 4). The cellular uptakes determined by flow cytometry of the nanoparticles showed cRGD-PSH-NP was effective in improving the transfection efficiency of survivin siRNA. The intracellular distribution of the nanoparticles was observed by confocal laser microscopy. The fluorescence signal of the group of cRGD-PSH-NP/S was obviously enhanced compared with the naked siRNA even in the presence of FBS. A large number of nanoparticles carrying siRNA surround and enter the cell, or even enter the nucleus which was more intuitive to observe that cRGD-PSH-NP/S was efficiently transfected into the tumor cells (Figures 5 and 6(A)). The expression level of survivin protein was determined to further evaluate the RNAi effect of siRNA. The survivin down regulations of cRGD-PSH-NP/S was 67.99%, which was significantly improved compared with PEI-NP/S and PSH-NP/S in HepG2 cells (Figure 6(B,C)).

*In vivo* antitumor efficacy of cRGD-PSH-NP/S showed that the tumor inhibition rate of cRGD-PSH-NP/S was 74.71% (Figure 7(B,C)), and the level of survivin in tumors treated with cRGD-PSH-NP/S was significantly lower than that treated with cRGD-NP/S (Figure 7(D)). Fortunately, the treatment did not affect the weight and safety of the nude mice (Figure 7(A) and 8). The *in vivo* and *in vitro* test results indicated that cRGD-PSH-NP was an effective and safe vector for loading and delivering siRNA into tumor cells to exert RNAi effect. The increase of the antitumor activity of cRGD-PSH-NP might be mainly due to the role of cRGD as a targeting ligand for the α_v_β_3_ integrin over-expression on the surface of hepatocarcinoma cells (Tang et al., 2017). At the same time, the reduction response characteristics and ‘proton pump effect’ of PSH were conducive to the disintegration of nanoparticles and the rapid release of siRNA into the cytoplasm (Yang et al., 2015). The long circulation characteristic of PEG-DSPE also played a certain role in the improvement of antitumor activity (Sasayama et al., 2019).

## 5. Conclusion

In conclusion, the nanoparticles cRGD-PSH-NP containing a cationic polymer based on PEI (PSH) and a targeting ligand (DSPE-PEG_2000_-cRGD) were efficient to deliver survivin siRNA. We showed that the cRGD-PSH-NP was low in toxicity and be able to bind siRNA efficiently. *In vitro* and *in vivo* experiments demonstrated that the cRGD-PSH-NP/S was safe and had high antitumor activity. We suggested that cRGD-PSH-NP might be suitable and effective for further research for the delivery of other nucleic acid drugs.
